# The burden of chronic ureteral stenting in cervical cancer survivors

**DOI:** 10.1590/S1677-5538.IBJU.2016.0667

**Published:** 2017

**Authors:** Yunhua Fan, Stephanie Jarosek, Sean P. Elliott

**Affiliations:** 1 Department of Urology, University of Minnesota, Minneapolis, Minnesota, USA

**Keywords:** Survivors, Neoplasms, Ureteral Obstruction

## Abstract

**Purpose:**

Ureteral obstruction in cervical cancer occurs in up to 11% of patients, many of whom undergo ureteral stenting. Our aim was to describe the patient burden of chronic ureteral stenting in a population-based cohort by detailing two objectives: (1) the frequency of repeat procedures for ureteral obstruction; and, (2) the frequency of urinary adverse effects (UAEs) (e.g., lower urinary tract symptoms, flank pain).

**Materials and Methods:**

From SEER-Medicare, we identified 202 women who underwent ureteral stent placement prior to or following cervical cancer treatment. The frequency of repeat procedures and rate ratios were compared between treatment modalities. The rates and rate ratios of UAEs were compared between our primary cohort (stent + cervical cancer) and the following groups: no stent + cervical cancer, stent + no cancer, and no stent + no cancer. The “no cancer” group was drawn from the 5% Medicare sample.

**Results:**

117/202 women (58%) underwent >1 stent procedure. The frequency of additional procedures was significantly higher in patients who received radiation as part of their treatment. UAEs were very common in women with stent + cancer. The rate of UTI was 190 (per 100 person-years), 67 for LUTS, 42 for stones, and 6 for flank pain. These rates were 3-10 fold higher than in the no stent + no cancer control group; rates were also higher than in the no stent + cancer and the stent + no cancer women.

**Conclusions:**

The burden of disease associated with ureteral stents is higher than expected and urologists should be actively involved in stent management, screening for associated symptoms and offering definitive reconstruction when appropriate.

## INTRODUCTION

Ureteral stents are integral to the management of many urologic and non-urologic conditions, however, pain, hematuria, bothersome urinary tract symptoms, infections, and encrustation are still common complications (1-3). While the morbidity of these adverse effects may be acceptable in temporary situations, like following ureteroscopy and lithotripsy, additional considerations are needed to measure the burden in patients requiring long-term stenting. Chronic ureteral stenting may be performed to manage various types of ureteral obstruction in which reconstruction is not feasible or not desired. The need for repeat stent exchanges, often every 3-6 months, and accompanied risk of anesthetic or iatrogenic complications, may cause a significant reduction in overall quality of life (4-6).

Ureteral obstruction in cervical cancer can be the result of disease progression, iatrogenic injury, or treatment toxicity with an incidence as high as 11% (7-10). With current estimates of approximately 245.000 cervical cancer survivors, there are significant patient and provider implications associated with chronic treatment of ureteral obstruction (11). Using a population-based cohort of women with non-metastatic cervical cancer and ureteral obstruction, we sought to describe the burden of chronic ureteral stenting by detailing two objectives: (1) the frequency of repeat procedures; and, (2) the frequency of urinary adverse effects (UAEs) such as lower urinary tract symptoms or flank pain. In order to estimate the contribution of cervical cancer and its treatment history vs. the contribution of having a ureteral stent on the frequency of UAEs we created several comparison groups. We compared the frequency of UAEs in women with cervical cancer and a stent to women with cervical cancer and no stent; we also compared the event rates to women without cancer who did or did not have a stent.

## MATERIALS AND METHODS

### Study Population

We have previously described how using the linked SEER-Medicare database we created a cohort of 1.808 women >66 years of age with non-metastatic cervical cancer diagnosed between 1992-2007 (10). Non-cancer controls were matched to cases 3:1 on birth year and race. These 5.424 controls were drawn from the 5% sample of Medicare beneficiaries residing in SEER areas with complete claims data and no history of pelvic malignancy.

From SEER, we obtained cancer subject’s basic demographic information including age at cancer diagnosis, race and ethnicity. Comorbidities were assessed by calculating the Charlson index from Medicare claims in the 12-months period before cancer diagnosis (12). Cervical cancer stage was determined based on the International Federation of Gynecology and Obstetrics (FIGO) staging system using the extension code and lymph node status from SEER. We identified primary cancer treatment as treatment received in the first 12 months after cancer diagnosis. Women were divided into 1 of 3 non-overlapping treatment groups: 1) External beam radiotherapy and brachytherapy (EBRT + BT), 2) Radiotherapy and surgery (RT + surgery), and 3) Surgery alone, to determine if specific treatment modalities were risk factors for requiring additional stent procedures. Patients were followed from the start of their cancer treatment to death or end of study period (Dec 31, 2009).

## OUTCOMES

### Primary objective

From the base cohort of 1808 women, we selected those who underwent at least one ureteral stent procedure in the 12 months prior to or at any time after cervical cancer treatment. All subsequent stent procedures were tallied as separate events. Ureteral stent procedures included stent placement, stent removal and, less commonly, nephrostomy tube placement and were identified using the respective ICD-9 procedure codes and CPT codes from MedPAR Inpatient, NCH and Outpatient Medicare claims data (see appendix). To avoid double counting, we required at least seven days between stent procedures. We described the demographic and clinical characteristics of the stented vs. non-stented cervical cancer cohort; comparisons were made using Student’s t-test or chi-square test, as appropriate.

We constructed a histogram of the total number of stent procedures performed per woman between the first procedure and end of the study period or death. Among cases who underwent stent removal, we separately described the frequency of nephrostomy placement. We did not assess the rate of stent change among the controls. We then accounted for differences in follow-up time by calculating the rate of stent procedures and compared rates and rate ratios across different cervical cancer treatment groups (EBRT + BT, Surgery + RT and Surgery). Poisson regression was performed to obtain multivariable-adjusted rate ratios, balanced for differences in demographic and clinical characteristics across treatment groups.

### Secondary objective

UAEs, as defined by the National Cancer Institute’s Common Terminology Criteria for Adverse Events were defined by a Medicare claim with an ICD-9 code corresponding to lower urinary tract symptoms, hematuria, incontinence, urinary retention, renal colic, urinary stones, or urinary tract infections (see appendix). We compared the rates of UAEs in our cohort of cancer cases with a stent (stent + cancer; n=202) to women with cancer but no stent (no stent + cancer; n=1606), controls with a stent (stent + no cancer; n=79), and controls without a stent (no stent + no cancer; n=5345). For women with a stent, UAEs were recorded from the time of initial stent placement in both the cancer cases (stent + cancer) and controls (stent + no cancer). In non-stented women, UAEs were recorded using a pseudo-diagnosis date based on FIGO stage in cases (no stent + cancer) and age-matching in controls (no stent + no cancer). UAEs were recorded from the initial time point through the end of study period or death. Specific UAEs were considered independently and each event could be counted more than once; however, to avoid double counting we required at least 7 days between claims for the same UAE. Rates of UAEs and multivariable-adjusted rate ratios are reported across the 4 groups, using Poisson regression. All statistical analyses were performed using SAS v9.3 (SAS Institute).

## RESULTS

From our initial population of 1808 women, we identified 202 (11.2%) who underwent at least one ureteral stent procedure in the 12 months prior to or any time after cervical cancer treatment. Among these 202 women (stent + cancer), there were a total of 540 stent procedures performed over 472 person-years. Primary cancer treatment was as follows: 117 were treated with EBRT + BT, 50 with Surgery + RT, and 35 with Surgery. The mean age was 73.7 years and was similar across treatment groups. Median follow-up was 2.5 years in the stent + cancer cohort compared to 4.3 years in the no stent + cancer group (p<0.0001). Advanced disease (FIGO stage III or IV) and death as endpoint were significantly more common in the stent + cancer patients compared to the no stent + cancer group (Table-1).

**Table 1:** Demographic and clinical characteristics of women with cervical cancer by ureteral stent.

	Stent + Cancer	No Stent + Cancer	p-value
No. of patients, n	202	1606	
Age at cancer diagnosis, mean (SD)	73.70 (5.64)	74.77 (6.35)	0.01
**Charlson Score, n (%)**			
0	137 (67.8%)	1009 (62.8%)	0.16
> 1	65 (32.2%)	597 (37.2%)	
**FIGO Stage, n (%)**			
1	51 (25.3%)	883 (55.0%)	<0.0001
2	58 (28.7%)	451 (28.1%)	
3-4 or Unknown	93 (46.0%)	272 (16.9%)	
Follow-up in years, Median (range, SD)	2.5 (0.04 to 16.2, 3.2)	4.3 (0.01 to 17.9, 4.0)	<0.0001
Death as endpoint, n (%)	154 (76.2%)	961 (59.8%)	<0.0001
**Treatment type, n (%)**			
EBRT + BT	117 (57.9%)	675 (42.0%)	<0.0001
RT + Surgery	50 (24.8%)	372 (23.2%)	
Surgery	35 (17.3%)	559 (34.8%)	

**Figure 1:**
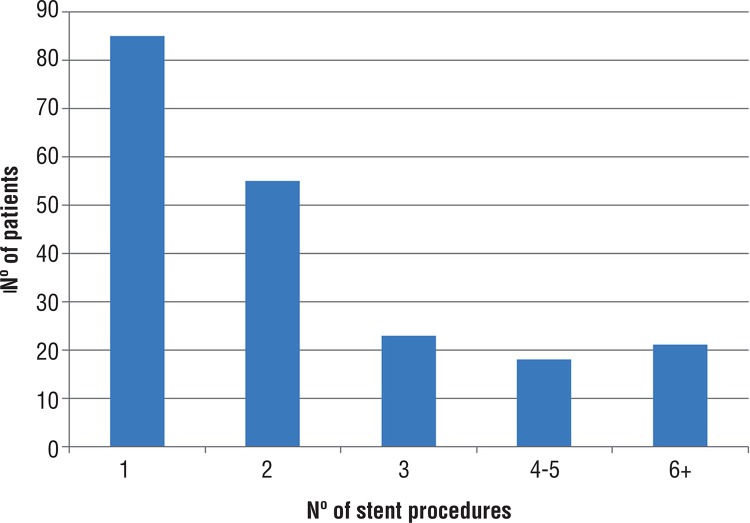
Number of stent procedures (initial and subsequent) performed in women with cervical cancer.

Of the 202 women, 85 (42%) underwent one stent placement procedure (no re-treatments), 55 (27%) were treated twice, and the remaining 62 (31%) were treated 3 or more times (Figure-1). 51 (25%) had the initial stent procedure in the 12 months prior to cancer treatment, 91 (45%) in the 12 months after treatment (including on the actual day of treatment), and 60 (30%) had the initial stent procedure more than 12 months after treatment. The most common initial procedure was cystoscopy with stent placement (57%, CPT 52332) followed by nephroureteral stent placement via percutaneous approach (20%, CPT 50393). Similar frequencies were observed for subsequent procedures as well. Stent removal without simultaneous replacement was performed in 80 patients, <11% of whom subsequently had a nephrostomy tube placed.

The rate of stent procedures was highest (1.54 per person-year) in the EBRT + BT group, followed by the Surgery + RT group (1.00 per person-year) and Surgery group (0.56 per person-year). After adjustment for age, race, FIGO stage and Charlson score, the RR was 2.40 (95% CI: 1.69-3.41) for EBRT + BT group and 1.81 (95% CI: 1.26-2.59) for Surgery + RT group, compared to Surgery group (Table-2).

**Table 2:** Rate and risk of stent procedures among the cervical cancer cases who underwent ureteral stent placement

	EBRT+BT	Surgery+RT	Surgery
No. of patients	117	50	35
No. of person-years	227.20	122.54	122.26
Unadjusted Rate* (95% CI)	1.54 (1.39-1.71)	1.00 (0.83-1.19)	0.56 (0.44-0.71)
Unadjusted RR* (95% CI)	2.77 (2.14-3.59)	1.79 (1.33-2.41)	1.00
Adjusted RR* (95% CI)	2.40 (1.69-3.41)	1.81 (1.26-2.59)	1.00

* Rates and rate ratios were obtained from Poisson regression. Adjusted rate ratios were further adjusted for age (65-69, 70-74, 75-79, 80-84, 85+), race (white, black, Hispanic, Asian, other/unknown), FIGO stage (1, 2, 3, 4, unknown), and Charlson comorbidity score (0, 1, 2+).

The rate (events/100 person-years) was determined for specific UAEs including lower urinary tract symptoms (LUTS), hematuria, incontinence, urinary retention, renal colic, urinary stones, or urinary tract infections (including pyelonephritis) and compared between groups (Table-3). The observed rate of UAEs in patients with stent + cancer was highest for UTIs (190), LUTS (67), and stones (42). The adjusted rate ratio (RR) was significantly higher in the stent + cancer group for all UAEs when compared to the no-stent + cancer group. With the exception of urinary stones, adjusted RRs were also higher in the stent + cancer group vs. the stent + no-cancer group, however, to a lesser extent (Table-4).

**Table 3:** Rate and risk of UAE among cervical cancer cases and controls.

	Controls (n=5424)		Cases (n=1808)	
	
	No cancer + no stent	No cancer + stent	Cancer + no stent	Cancer + stent
No. of patients	5,345	79	1606	202
No. of person-years	45,917.90	701.11	7360.89	472.00
Unadjusted Rate* (per 100 person-year)				
Lower Urinary Tract Symptoms	17.9	36.5	24.7	67.2
Hematuria	1.24	4.43	2.67	7.42
Incontinence	6.73	21.99	8.41	20.97
Retention	0.95	1.95	0.73	7.84
Renal Colic/Flank pain	0.58	3.90	0.65	5.93
Stones	1.75	88.67	1.54	41.53
UTI/Pyelonephritis	51.02	113.15	58.36	190.48
**Adjusted RR** ^**†**^ **(95% CI)**				
Cystitis and Spasm	1.00	1.86 (1.62-2.14)	1.37 (1.29-1.44)	3.80 (3.40-4.27)
Hematuria	1.00	3.13 (2.08-4.71)	2.12 (1.79-2.52)	5.87 (4.15-8.30)
Incontinence	1.00	2.84 (2.37-3.42)	1.23 (1.12-1.34)	3.17 (2.59-3.88)
Retention	1.00	1.99 (1.08-3.65)	0.76 (0.57-1.01)	8.48 (6.02-11.94)
Renal Colic/Flank pain	1.00	6.02 (3.82-9.49)	1.07 (0.78-1.48)	10.22 (6.85-15.24)
Stones	1.00	48.14 (42.38-54.68)	0.85 (0.70-1.05)	24.57 (20.86-28.94)
UTI/Pyelonephritis	1.00	1.80 (1.66-1.95)	1.12 (1.08-1.16)	3.75 (3.50-4.01)

**Table 4:** Rate ratios of UAE among cervical cancer cases and controls.

	Controls (n=5424)		Cases (n=1808)	
	
	Controls without stent	Controls with stent	Cases without stent	Cases with stent
**Adjusted RR** ^*^ **(95% CI)**				
Cystitis and Spasm	-	1.00	-	2.05 (1.72-2.45)
Hematuria	-	1.00	-	1.88 (1.12-3.14)
Incontinence	-	1.00	-	1.11 (0.85-1.45)
Retention	-	1.00	-	4.26 (2.16-8.39)
Renal Colic/Flank pain	-	1.00	-	1.70 (0.96-2.99)
Stones	-	1.00	-	0.51 (0.43-0.61)
UTI/Pyelonephritis	-	1.00	-	2.08 (1.88-2.30)
**Adjusted RR** ^*^ **(95% CI)**				
Cystitis and Spasm	-	-	1.00	2.79 (2.48-3.14)
Hematuria	-	-	1.00	2.76 (1.93-3.96)
Incontinence	-	-	1.00	2.58 (2.09-3.19)
Retention	-	-	1.00	11.21 (7.37-17.04)
Renal Colic/Flank pain	-	-	1.00	9.53 (5.97-15.21)
Stones	-	-	1.00	28.76 (22.82-36.25)
UTI/Pyelonephritis	-	-	1.00	3.35 (3.12-3.60)

* Adjusted rate ratios were obtained from Poisson regression, with further adjustment for age (65-69, 70-74, 75-79, 80-84, 85+), race (white, black, Hispanic, Asian, other/unknown), and Charlson comorbidity score (0, 1, 2+).

## DISCUSSION

Quantifying the burden of chronic ureteral stenting is important to improve the quality of life of cancer survivors. This is particularly important in cervical cancer because ureteral obstruction occurs in 11%. We show that over 50% of the women who had a ureteral stent placed underwent an additional stent procedure, and over 30% underwent 3 or more procedures. We also show that the incidence of UAEs in women who underwent stent placement was significantly higher than cervical cancer patients without stents as well as control patients with stents.

Ureteral stenting in patients with cervical cancer may be necessary for disease progression (i.e., malignant ureteral obstruction) or for ureteral stricture occurring as an adverse effect of cancer treatment. While malignant obstruction is associated with a median survival of only 3-6 months, ureteral stricture often becomes a chronic medical problem throughout the cancer survivorship phase (13, 14). Because of complexities in the multi-disciplinary management of patients with non-urologic malignancies, urologists may be removed from ureteral obstruction management decisions; in some centers the gynecologic oncologist may consult interventional radiology instead. Our findings support the conclusion that urologists should be actively involved in the management of these patients to manage stent-related side effects, monitor for stent failure or decline in renal function, and offer definitive reconstruction when indicated.

The morbidity related to ureteral stents has been well described. Possible complications include pain, voiding symptoms, bleeding, infections, encrustation, and the potential to be forgotten (15, 16). Because of the proximity of the cervix to the bladder and other urologic structures, treatment of cervical cancer is associated with many urinary side effects irrespective of stenting (17, 18). We hypothesized that patients with both cervical cancer and a ureteral stent would have higher rates of UAEs than each group separately; however, the rate ratios were more extreme than we expected. The only UAE that was not more common in the stent + cancer group was urinary stones; this was most common in the stent + no cancer group where calculi (renal or ureteral) was the indication for stent placement in over 50% of the patients.

Some limitations of our study deserve mention. First, we cannot know the exact reason why a stent was placed. There was significant variation in the timing of stent placement and treatment, suggesting differences in the etiology of ureteral obstruction and the likelihood of resolution. Still, by excluding women with metastatic disease and limiting our analysis to those with a diagnosis of ureteral obstruction or ureteral stricture, we have shown that there is a sensitivity of 100% and specificity of 99% for detecting ureteral strictures after cervical cancer treatment (19). Second, we don’t know if a stent was placed on a different side or in conjunction with a minimally invasive procedure to treat the obstruction. We did observe that very few patients received definitive reconstruction (less than 5%); better integration of urologists in the management of these women may help get them access to reconstructive options. Third, we cannot know whether the stent was the cause of the UAEs, only that stent placement was highly associated with ureteral stenting. Because stenting was so highly correlated with advanced stage cancer and treatment with radiotherapy, we could not isolate the effect of the stent from these other factors in multivariate models. Finally, the patient characteristics and outcomes observed in this Medicare population may differ from the experience in other groups of cervical cancer patients.

## CONCLUSIONS

Ureteral stents may represent the only long-term treatment option for certain patients with ureteral obstruction. Furthermore, the degree of urinary adverse effects in patients with ureteral stents may vary considerably between different populations. In our study, women with ureteral stents treated for cervical cancer had significantly higher rates of UAEs compared to patients with ureteral stents without cancer. Because of the complexity involved in stent management, including coordination of exchanges, assessment of adverse effects, and continual consideration for definitive reconstruction, urologists should remain actively involved in the care of such patients.

## ARTICLE INFO

Int Braz J Urol. 2017; 43: 104-11
